# Risk Assessment of Depression amongst Women during Menopause before and during the COVID-19 Pandemic

**DOI:** 10.3390/ijerph20010596

**Published:** 2022-12-29

**Authors:** Agnieszka Kułak-Bejda, Katarzyna Krajewska-Ferishah, Agnieszka Szyszko-Perłowska, Napoleon Waszkiewicz

**Affiliations:** 1 Department of Psychiatry, Medical University of Białystok, 15-272 Białystok, Poland; 2 Department of Integrated Medical Care, Medical University of Białystok, 15-096 Białystok, Poland

**Keywords:** menopause, depression, 2006, 2021

## Abstract

During menopause, the risk of depression is 2–5 times greater than pre- or post-menopause. To assess the risk of depression amongst menopausal women in 2006, compared with 2021 (i.e., during the COVID-19 pandemic), we recruited female volunteers at least 40 years old to complete the menopause rating scale (MRS), the Blatt–Kupperman menopausal index (BKMI), and the Beck depression inventory. Compared with 2006, in 2021, the number of participants who experienced mild, moderate, and severe menopausal symptoms rose by 8%, 1.9%, and 3.2%, respectively. Moreover, the frequency of using hormone replacement therapy rose by 5.1%, feelings of mistrust by 16%, feelings of guilt by 11%, disposition to cry by 25%, mood swings by 12%, and suicidal ideation by 9%, whilst the average body weight and body mass index fell by 5.1 kg and 2.3%, respectively. In 2006 and 2021, 46.3% and 59.3% of participants experienced moderate depression, whereas 3.0% and 5.2% experienced severe depression. The number of participants who denied needing support during menopause decreased by 14.6%, whereas the demand for support from husbands and friends increased by 18.3% and 9.8%, respectively. In 2021, the number of participants who experienced menopausal symptoms, a loss of trust, self-blame, a tendency to cry, mood swings, suicidal ideation, and anxiety also increased compared with 2006, whilst the number of participants without depressive symptoms decreased, but with suspected mild or severe depression increased. Last, menopausal complaints caused an increase in depressive mood in 2021, as evaluated using the BKMI and MRS.

## 1. Introduction

Menopause, a climacteric change in life, is usually a biological process in which the oestrogen production in women gradually decreases before ultimately ceasing, as initially manifested by the irregularity of menstrual cycles until their complete cessation. Vasomotor symptoms, including hot flashes and night sweats, are the most common symptoms of menopause and affect more than 80% of women during their periods, whilst other common symptoms include disturbed sleep, fatigue, depressed mood, brain fog, low libido, and heightened anxiety [1].

During menopause, the risk of depression is 2–5 times greater than before menopausal changes first appear and after menopause ends [2,3]. Amongst women experiencing menopause, the development of depression that requires pharmacological therapy largely depends on their age at which menopause occurs and is more frequent if climacteric symptoms occur before the age of 45 years (10–15%), but less frequent amongst women whose menopause begins at the age of 48 years or later (5–6%) [4,5]. Research has additionally revealed an association between depressive and menopausal symptoms and the involvement of gonadal hormones, cortisol, and serotonin deficiency in the process [6]. The risk of developing depression is also greater amongst women previously diagnosed with depressive disorders requiring antidepressants [7], as well as identified as having premenstrual syndrome, depression during pregnancy or in the postpartum period, dysphoric mood states caused by taking oral contraceptives and postpartum blues. The risk of depression amongst menopausal women is also greater, due to intensified negative experiences with lifestyle changes in motherhood, family life, professional life, fertility, physical fitness, and attractiveness, and amongst women who excessively worry about others [5,7].

Furthermore, since early 2020, the risk of depression has been exacerbated by the COVID-19 pandemic and related worries about personal and loved ones’ health, the threat of unemployment, and household finances, along with constraints on socialising, confinement, boredom, a longing for active leisure, and exhaustion as a result of the prolonged health, economic, and social crises [8].

Against that background, the aim of our study was to compare the risk of depression amongst women during menopause in 2006 and in 2021 (i.e., during the COVID-19 pandemic).

## 2. Material and Methods

Our study was conducted with the approval of the Bioethics Committee of the Medical University in Białystok (No. R-I-003/118/2006 for the 2006 data and No. APK.002.587.2021 for the 2021 data). Although data were collected from a different sample of women in 2006 vs. 2021, identical inclusion and exclusion criteria were applied in both years: being female, being at least 40 years old, participating in the study voluntarily, and completing all questionnaires. Table 1 details the socio-demographic data of the participants.

In both years, the same three questionnaires were administered in a single survey: the menopause rating scale (MRS), the Blatt–Kupperman menopausal index (BKMI), and the Beck depression inventory (BDI). First, the MRS, developed by Lothar A. J. Heinemann of the Centre for Epidemiology and Health Research in Berlin, has been recognised as a valid instrument for measuring health-related quality of life, one that is easy for participants to complete [9]. The purpose of the MRS is to compare women with different conditions, regarding their symptoms and the severity of their symptoms, and to assess changes before and after treatment. With a simple scoring system, it consists of 11 questions about the presence of hot flashes and sweating, chest discomfort, sleep disturbances, depressive symptoms, irritability, anxiety, physical and mental exhaustion, muscle and joint discomfort, sexual problems, urinary problems, and vaginal dryness. Women rate the severity of experiencing each complaint on a 5-point scale (i.e., 0 = *no symptoms*, 1 = *mild*, 2 = *moderate*, 3 = *severe*, 4 = *very severe*), for a maximum of 44 points, indicating the span and severity of changes, from 0 (i.e., no symptoms) to 44 points (i.e., all symptoms and numerous complaints). However, the minimum and maximum number of points varies between three different dimensions: psychological symptoms range from 0 to 16 points, with four symptoms (i.e., depression, irritation, anxiety, and exhaustion); somatic–vegetative symptoms range from 0 to 16 points, also with four symptoms (i.e., sweating and hot flashes, chest complaints, sleep disturbances, and joint and muscle pain); and complaints regarding the genitourinary system range from 0 to 12 points, with three symptoms (i.e., sexual problems, complaints regarding the urinary system, and vaginal dryness). In all cases, the higher the score, the greater severity of the climacteric symptoms [9]. The internal consistency of the MRS, measured with Cronbach’s alpha, revealed coefficients ranging between 0.6 and 0.9 across countries for total scores, as well as scores in the three domains. In contrast, the test–retest coefficients of total scores ranged from 0.80 to 0.96 across Europe, North American, Central America, and Asia.

Second, to assess the severity of climacteric symptoms, we employed the BKMI, which has patients rate the severity of 11 complaints (i.e., hot flashes, excessive sweating, sleep disturbances, excessive nervousness, depressed mood, dizziness, lack of energy, joint pain, headaches, cardiac arrhythmia, and paraesthesia) on a 4-point scale [10,11]. Scores less than or equal to 20 points indicate no climacteric symptoms, scores of 21–25 points indicate mild symptoms, scores of 26–30 points indicate moderate symptoms, and scores greater than 30 points indicate severe symptoms. The internal consistency of the BKMI, measured with Cronbach‘s alpha, shows coefficients ranging from 0.92 to 096, whilst the 1-week test–retest consistency was 0.93 [10,11].

Third and last, the BDI distinguishes four states of depression’s severity—no depression, mild, moderate, and severe depression—on the previous day [12]. The scale consists of 21 questions, each with three answer options and each is scored differently, depending on the answer given. Depending on the scores, the BDI provides a diagnosis: 0–11 points for no depression or depressed mood, 12–27 points for moderate depression, and 28 or more points for severe depression [12].

The statistical analysis of the collected data was performed using a descriptive method and by testing correlations between quantitative and qualitative characteristics in the data. If a given characteristic was numerical, then the values of the descriptive statistics in each country were determined, and the *t*-test and Wilcoxon test were applied. Spearman‘s rank coefficient of correlation was used. For qualitative characteristics, the percentage of responses by country was determined, along with the result of the chi-squared test of independence with Yates’ correction. Spearman’s rank correlation test was used to assess the relationships between the BKMI and the MRS. All data and statistical analyses were performed in Statistica PL (version 13.0), and *p*-values less than 0.05 were considered to indicate statistical significance.

## 3. Results

Amongst the participants—all women from Poland—from 2006 to 2021, the average body weight dropped significantly (*p* < 0.001)—namely, from 70.5 ± 11.6 kg (range: 40–125) to 65.4 ± 8.2 kg (range: 40–98)—whereas the average height rose, from 162.8 ± 5.4 cm (range: 140–178) to 165.2 ± 6.2 cm (range: 150–180), but not significantly. Their average body mass index in 2006 was 26.6 ± 4.0% (range: 17.8–41.3), which indicates overweight, but in 2021, it was 24.3 ± 3.2% (range: 19.2–30.2), which indicates normal weight (*p* < 0.03). The average age at menarche—14.0 ± 1.4 years in 2006 and 14.0 ± 2.3 years in 2021—did not differ significantly, whereas the average fertility rate did: from 2.5 ± 1.0 (range: 1–5) in 2006 to 2.2 ± 0.9 (range: 1–4) in 2021 (*p* < 0.001). In 2006, hormone replacement therapy (HRT) was used by 54.8% of the women, but by 59.9% of them in 2021 (n.s.). No significant correlations between the HRT and the BDI in 2006 (r = 0.120; *p* = 0.223) and 2021 (r = 0.141 *p* = 0.328) were found. BKMI scores indicated no climacteric symptoms in 71.8% of participants from Poland in 2006, compared with 58.8% in 2021, which marked a significant change (*p* < 0.001). Moreover, in 2006 vs. 2021, 18.5% and 26.5% of participants experienced mild depressive symptoms (n.s.), 6.7% and 8.6% experienced moderate symptoms (n.s.), and 2.9% and 6.1% experienced severe symptoms (n.s.), all respectively.

The average BKMI score amongst all participants in 2006 was 14.8, which indicates no menopausal symptoms, but 20.1 points in 2021, which generally indicates mild severity (n.s.). Meanwhile, the average overall MRS score for all participants, out of a possible 44 points, was 12.2 points in 2006 and 22.8 points in 2021. As for the three types of symptoms covered by the MRS, the average score for psychological symptoms, out of a possible 16 points, was 4.8 points in 2006, but 9.4 in 2021; the average score for somatic–vegetative symptoms, also out of a possible 16 points, was 4.9 points in 2006 but 10.4 in 2021; and the average scores for genitourinary system symptoms, out of a possible 12 points, were 2.5 points in 2006 and 5.2 points in 2021. In 2006 vs. 2021, 18.5% vs. 26.5% of participants showed mild menopausal symptoms, 6.7% vs. 8.6% showed moderate symptoms, and 2.9% vs. 6.1% showed severe symptoms, all respectively. All other data are shown in Table 2.

In 2006 vs. 2021, the average points scored, according to the BDI, were 10.5 ± 7.9 (range: 0–39) vs. 23.8 ± 9.7 (range: 0–48), respectively (*p* < 0.001). Mild depression was found amongst 36.3% of participants in 2006, but amongst 59.3% in 2021, whilst severe depression was found amongst 3.0% of participants evaluated in 2006, but amongst 5.2% in 2021. Menopausal complaints, as evaluated using the BKMI and MRS, caused an increase in depressive moods from 2006 to 2021, as shown in Table 3. By contrast, Table 4 presents the differences in the incidence of the symptoms accompanying menopause in 2006 vs. 2021.

Differences in the incidence of symptoms accompanying menopause in 2006 and 2021 are shown in Table 4.

Table 5 shows the list of people from whom participants most expected support. In 2006 vs. 2021, the number of participants who denied needing support during menopause decreased from 23.7% to 9.6%, respectively, whilst the demand for support from husbands increased from 60.6% to 78.9%, and the demand for support from friends from 30.7% to 40.5%.

## 4. Discussion

In the present study, the percentage of women without menopausal symptoms, according to the BKMI in 2021 (58.8%), was significantly lower than in 2006 (71.8%). However, the percentage of women with moderate depression, according to the BDI, was significantly higher in 2021 (59.3%) than in 2006 (36.3%). Symptoms accompanying menopause (e.g., loss of confidence, guilt, tendency to cry, suicidal ideation, and attention deficit) were reported significantly more often by women in 2021 than in 2006. Furthermore, in 2021, women with menopause reported receiving significantly less support from friends of both genders (6.8%) than in 2006 (12.0%), as well as significantly less support from general practitioners (4.9% vs. 17.8%). 

The current results are consistent with some findings from previous studies [13,14,15,16,17] and justify growing awareness of the fact that psychological symptoms may play a prominent role during menopause and may even dominate the period’s clinical profile. For example, in a study conducted in China [13], the prevalence of symptoms of anxiety and depression was higher in the year of final menstruation than in the year before; however, the prevalence of such symptoms in the early postmenopausal years was even higher. Studies conducted in the United States have also shown that socio-economic and health-related factors influence the course of depressive symptoms [14,15]. Another study conducted in both Australia and Japan showcased the importance of sleep problems, low social support, life events, low levels of physical activity, and low role function as risk factors for long-term depressive symptoms amongst women [16].

In a study conducted in the United States [17], approximately half of participating women reported unspecific anxiety (e.g., persistent premenstrual symptoms) during menopause. Other findings indicated that women with depression, anxiety, and generally negative mood were at increased risk of reporting vasomotor symptoms. However, vasomotor symptoms can also negatively impact mood. In a study conducted with middle-aged menopausal women in South Korea [18], the incidence of suicidal ideation amongst women with primary ovarian insufficiency, at upwards of 20%, was far higher than in the general population. In addition, age at menopause was found to be associated with the incidence of suicidal ideation and poor self-esteem amongst women not diagnosed with major depressive disorder.

According to a systematic review of 14 studies involving a total of 67,714 women in the United States, Turkey, Israel, and various European countries, increased age at menopause (i.e., in 2-year increments) was associated with a 2% decrease in the risk of depression during menopause [19]. Moreover, an accompanying meta-analysis of four studies involving 3033 women showed that primary ovarian insufficiency was associated with a twofold-greater risk of depression than menopause at an age of at least 40 years.

Several mechanisms may explain the association between primary ovarian insufficiency and depressive symptoms. It has been suggested that the prolonged deprivation of oestrogens, which exert neuroprotective effects via receptors in the brain, amongst women with primary ovarian insufficiency may have adverse effects on mood disorders [20,21]. A younger age at menopause may also indicate shorter exposure to the neuroprotective and anti-depressive effects of endogenous oestrogens. Beyond that, low serotonergic activity is associated with hypo-oestrogenism and appears to increase the risk of suicide amongst individuals with predisposing factors. On that count, a somewhat worrying result of our study is that the rate of suicidal ideation increased by 9% from 2006 to 2021. 

According to data from the World Health Organization (WHO), as of 7 July 2021, 184,324,026 confirmed cases of COVID-19 had been reported to the WHO, including 3,992,680 deaths [22], most of which occurred in North and South America, not in Europe or Southeast Asia. However, the highest excess of deaths were recorded in the United States, Italy, England, and Wales, Spain, and Poland [23]. Although Poland, similarly to the Czech Republic, Slovakia, Hungary, Denmark, Finland, Bulgaria, and Australia, avoided a detectable increase in mortality, for whatever reason, in the first wave of the COVID-19 pandemic (i.e., the end of May 2020), they did not in the second half of 2020. Based on our results, Poland is a country where excessive mortality has been observed throughout the pandemic, compared with the number of deaths in 2019–2020 only. 

An international study on the COVID-19 pandemic’s influence on suicides worldwide from mid-February through May 2020 was conducted by researchers at the International COVID-19 Suicide Prevention Research Collaboration [24]. England and Wales, as well as Spain, demonstrated the greatest level of influence. By comparison, Bulgaria, New Zealand, Slovakia, Australia, the Czech Republic, Hungary, Poland, Norway, Denmark, and Finland experienced changes in mortality ranging from small declines to increases of 5% or less. Meanwhile, a retrospective 4-year study analysing changes in suicide rates in Nepal during the COVID-19 pandemic (i.e., from April 2020 to June 2021) vs. before the pandemic counted 24,350 suicides and showed a general increase in the monthly suicidal rate, with an average increase of 0.28 suicides per 100,000 people during the pandemic [25].

In other work, Calati et al.’s study [26] on the evaluation of suicidal rates in Lombardy, Italy, was based on the retrospective analysis of all autopsies conducted in 2020 and in the first four months of 2021. In 2020, the number of recorded suicides decreased, compared with rates in 2016–2019 (i.e., 21.19–22.97% of autopsies), and amounted to 98 (i.e., 18.08% of 542 autopsies), compared with 35 suicides in the first 4 months of 2021 alone (i.e., 185 autopsies total) [20]. Likewise, a study conducted in Poland that involved analysing the reports of the Ministry of Health, the reports of the Social Insurance Institution, and police statistics revealed an increase (26.86%) in the number of deaths in 2021 vs. in 2017–2019 [27]. 

The mentioned trends seem to relate to WHO data from the first year of the COVID-19 pandemic, when the global frequency of anxiety and depression increased by as much as 25% [28]. The WHO’s data are based on a review from 5683 sources (e.g., PubMed and Google Scholar), addressing the spread of major depressive and anxiety disorders during the pandemic, published between 1 January 2020 and 29 January 2021 [29]. Two influential indicators of COVID-19, namely the daily SARS-CoV-2 infection rate and the decrease in people’s mobility, related to the increased frequency of major depression (regression coefficient [B] = 0.9 [95% uncertainty range: 0.1–1.8; *p* = 0.029] for individual mobility and B = 18.1 [7.9–28.3; *p* = 0.0005] for daily SARS-CoV-2 infection) and of anxiety disorders (B = 0.9 [0.1–1.7; *p* = 0.022] for individual mobility and B = 13.8 [10.7–17.0; *p* < 0.0001 for daily SARS-CoV-2 infection]). Women were more affected than men (B = 0.1 [0.1–0.2; *p* = 0.0001 for major depression and B = 0.1 [0.1–0.2; *p* = 0.0001] for anxiety disorders), and the younger age groups were affected by both major depression and anxiety disorders. Another 53.2 million cases (range: 44.8–62.9) of major depression have been estimated globally—an increase of 27.6%—due to the pandemic. Thus, the overall incidence of depression amounted to 3152.9 cases (range: 2722.5–3654.5) per 100,000 inhabitants. Added to that, 76.2 million cases of anxiety disorders have been estimated globally, for an increase of 25.6%. From another perspective, major depressive disorders have resulted in an average of 49.4 million (range: 33.6–68.7 million) disability-adjusted life years (DALYs) and anxiety disorders 44.5 million (range: 30.2–62.5 million) DALYs globally in 2020. In a cross-sectional observational study conducted from February to April 2021, Dankowski et al. [30] tested patients who had had COVID-19 at least 28 days after their diagnosis. Symptoms reported by the patients were assessed using, amongst other things, the BDI and the state–trait anxiety inventory. The participants included 10,245 men (44%), and they were 52 ± 13 years old on average. Mild depressive symptoms were observed in nearly 30% of patients. Moreover, scores were significantly higher in women than in men. In our study, anxiety increased by 15% in 2021, compared with 2006, and the average level of points obtained using the BDI increased by 13.3%. We also found that the number of women without depressive symptoms decreased by 25.2%, the number of women with suspicion of moderate depression increased by 23%, and the number of women with severe depression increased by 2.2%.

The results of studies by Folkman and Lazarus [31] and Coyne [32] indicate the severity of depressive disorders increases with the increased distance in social contact with other people. In our study, the number of women who denied needing support during menopause decreased in 2021 vs. 2006 by 14.6%. However, the demand for support from husbands and friends increased by 18.3 and 9.8%, respectively.

In 2021, the number of participants who experienced menopausal symptoms and a significant loss of confidence, self-blame, a tendency to cry, mood swings, and a higher level of anxiety and suicidal ideation increased, compared with 2006, whereas the number of women without depressive symptoms decreased, and the suspicion of mild and severe depression increased. Menopausal complaints caused an increase in depressive moods in 2021 vs. 2006, as evaluated using the BKMI and MRS, and the demand for support from husbands and friends increased, as well. 

Amongst other results, most women (57%) had heard of menopausal hormone therapy, and nearly one in five participants (19%) had used it themselves. Beyond that, the frequency of using HRT increased by 5.1% in 2021, compared with 2006. Amongst fears related to menopause, participants in 2021 mentioned increased body weight (34%), the sudden appearance of symptoms (e.g., hot flashes, dizziness, and fainting; 33%), and malaise (e.g., depression, irritability, and anxiety; 30%). A decrease in average body weight was also recorded from 70.5 kg in 2006 to 65.4 kg in 2021. 

In a study conducted in 2022, 29% of participants experiencing menopause declared that the COVID-19 pandemic had worsened their ability to deal with menopausal symptoms. The participating women most frequently reported fatigue, sleep disturbances (43%), weight gain (40%), low well-being (31%), and headache (29%) [28]. In our study, the loss of trust increased by 16% from 2006 to 2011, along with feelings of guilt (i.e., by 11%) and the tendency to cry (i.e., by 25%).

### 4.1. Strengths

Amongst our study’s strengths, the sample was homogeneous, Moreover, we assessed the risk of depression amongst women during menopause at two time points—in 2006 and in 2021 (i.e., during the COVID-19 pandemic)—by using three questionnaires. In addition, many risk factors for depression amongst women during menopause were analysed. 

### 4.2. Limitations

One of our study’s limitations was the low number of women studied. Moreover, we did not use multivariate statistics to determine the relationships between variables. We also assessed the risk of depression during the COVID-19 pandemic, which may have increased the incidence of depression, anxiety, and menopausal symptoms in the sample. Last, we did not analyse the effect of HRT anxiety or suicidal ideation. In future research, it would be interesting to use depression scales other than the BDI. The BDI limitations include lack of representative norms, and thus, doubtful objectivity of interpretation, controversial factorial validity, instability of scores over short time intervals (over the course of 1 day), and poor discriminant validity against anxiety.

## 5. Conclusions

In 2021 vs. 2006, both the average body weight and body mass index of participating women decreased, whereas the frequency of using HRT rose. The number of participants who experienced menopausal symptoms and an increased loss of trust, self-blame, tendency to cry, mood swings, anxiety, and suicidal ideation also rose, as did the proportion of women who suspected having mild or severe depression. Per the BKMI and MRS scores, menopausal complaints caused an increase in depressive mood in 2021, compared with 2006, during which time, the demand for support from husbands and friends increased, as well.

## Figures and Tables

**Table 1:** Socio-demographic data of women.

Variables	Year 2006	Year 2021
Number of females (N)	241	350
Age (years, mean ± SD)	50.7 ± 4.3	51.5 ± 4.1
Living in the big city	32.8%	41.2%
Living in the small towns	47.7%	39.5%
Living in the villages	19.5%	7.9%
Higher education	21.2%	45.6%
Secondary education	53.5%	31.4%
Vocational education	17.4%	18.5%
Primary school education	7.9%	4.5%
Very good economic situation	3.7%	12.1%
Good economic situation	34.4%	42.1%
Average economic situation	56.0%	42.6%
Bad economic situation	5.8%	3.2%

**Table 2:** The scale of menopausal symptoms in 2006 and 2021.

The Blatt–Kupperman Index					
	$\bar{x}$	*s*	Min	Max	*p* value
Year 2006	14.8	8.6	0	41	<0.001 Wilcoxon test
Year 2021	20.1	8.3	0	45	
Wilcoxon test	no symptoms	mild severity	moderate severity	serious severity	# *p* < 0.05 Chi-squared test
Year 2006	71.8%	18.5%	6.7%	2.9%	
Year 2021	58.8% #	26.5%	8.6%	6.1%	
	$\bar{x}$	*s*	Min	Max	*p* value Wilcoxon test
Total MRS					
Year 2006	12.2	7.6	0	37	<0.001
Year 2021	22.8	6.5	0	44	
MRS—psychological symptoms					
Year 2006	4.8	3.4	0	16	<0.001
Year 2021	10.4	4.2	0	16	
MRS—somatic-vegetative symptoms					
Year 2006	4.9	3.0	0	13	<0.001
Year 2021	7.2	3.1	0	16	
MRS—symptoms on the part of the genitourinary system					
Year 2006	2.5	2.4	0	11	<0.001
Year 2021	5.2	2.8	0	12	

**Table 3:** The severity of depressive symptoms, according to the Beck scale, in 2006 and 2021.

Years	The Severity of Depression, According to the Beck Inventory					
	No Depression	Moderate Depression		Severe Depression		*p* Value Chi-Squared Test
2006	60.7%	36.3%		3.0%		*** *p* < 0.001 * *p* < 0.05
2021	35.5% ***	59.3% *		5.2%		
		2006		2021		*p* value Wilcoxon Test
		$\bar{x}$	Me	$\bar{x}$	Me	
Education						
primary		17.4	17.0	17.6	19.0	NS
vocational		12.8	12.0	5.9	6.0	NS
secondary		9.0	7.5	22.2	10.5	<0.001
higher		9.8	9.0	19.7	9.0	<0.001
Taking HRT						
yes		12.1	11.0	12.3	9.0	NS
no		10.2	9.5	14.9	11.0	<0.001
The Blatt–Kupperman Index						
Spearman’s rank coefficient of correlation		r = 0.47		r = 0.54		NS
MRS scale						
Spearman’s rank coefficient of correlation		r = 0.68		r = 0.78		NS

Me-median.

**Table 4:** Symptoms accompanying menopause in 2006 and 2021.

Issue	2006			2021		
	Never	Sometimes	Often	Never	Sometimes	Often
loss of confidence	69%	26%	5%	32% ***	47% ***	21% ***
anxiety	52%	39%	10%	10%	62%	25%
guilty	49%	39%	12%	27% ***	45% **	23% ***
tendency to cry	37%	45%	18%	27% ***	56% ***	43% ***
suicidal thoughts	89%	10%	2%	29% ***	29% ***	11% ***
mood swings	17%	63%	19%	18%	69%	31% *
decrease in libido	38%	44%	18%	42%	29%	8% **
attention deficit	37%	56%	7%	37%	34% **	21% ***
trouble with memory	33%	55%	13%	46% *	25% ***	11%
dryness of mucous membranes	62%	30%	9%	36%	31%	10%
dysgeusia	90%	8%	3%	28% ***	9%	0% ***
dryness of the vagina	48%	41%	11%	23% **	33%	8%
urinary tract infection	67%	26%	7%	36% ***	19%	5%
urinary incontinence	62%	27%	12%	29% ***	18%	5%
vaginitis	71%	24%	5%	44% ***	20%	3%
painful intercourse	59%	31%	10%	36% **	25%	6%

Chi-squared test: *** *p* < 0.001 2006 vs. 2021; ** *p* < 0.01 2006 vs. 2021; * *p* < 0.01 2006 vs. 2021.

**Table 5:** The list of persons from whom climacteric women expect support.

They Expect Support from ...	2006	2021	Chi-Squared Test
husband	60.6%	78.9%	NS
friends	30.7%	40.5%	NS
male friends/female friends	12.0%	6.8%	<0.05
midwives/nurses	8.3%	5.4%	NS
gynaecologist	22.0%	19.8%	NS
psychologist	5.8%	4.9%	NS
GP	17.8%	5.5%	<0.001
no expectation	23.7%	9.6%	<0.001

## Data Availability

Data of this study are available on the request.
